# Gastrointestinal Opportunistic Infections in Human Immunodeficiency Virus Disease

**DOI:** 10.4103/1319-3767.48965

**Published:** 2009-04

**Authors:** Awadh R. Al Anazi

**Affiliations:** Department of Medicine (38), King Khalid University Hospital, College of Medicine, King Saud University, P. O. Box 2925, Riyadh, 11461, Saudi Arabia

**Keywords:** Gastrointestinal opportunistic infections, highly active antiretroviral therapy, HIV disease

## Abstract

Gastrointestinal (GI) opportunistic infections (OIs) are commonly encountered at various stages of human immunodeficiency virus (HIV) disease. In view of the suppressive nature of the virus and the direct contact with the environment, the GI tract is readily accessible and is a common site for clinical expression of HIV. The subject is presented based on information obtained by electronic searches of peer-reviewed articles in medical journals, Cochrane reviews and PubMed sources. The spectrum of GI OIs ranges from oral lesions of Candidiasis, various lesions of viral infections, hepatobiliary lesions, pancreatitis and anorectal lesions. The manifestations of the disease depend on the level of immunosuppression, as determined by the CD4 counts. The advent of highly active antiretroviral therapy has altered the pattern of presentation, resorting mainly to features of antimicrobial-associated colitis and side effects of antiretroviral drugs. The diagnosis of GI OIs in HIV/ acquired immunodeficiency syndrome patients is usually straightforward. However, subtle presentations require that the physicians should have a high index of suspicion when given the setting of HIV infection.

The acquired immunodeficiency syndrome (AIDS) is caused by the human immunodeficiency virus (HIV). The disease has significantly changed medical practice globally.[1] The HIV/AIDS epidemic has already claimed more than 25 million lives and another 40 million people were estimated to be living with HIV/AIDS worldwide by the end of December 2005.[2] National data analyzed between 1984 and 2003 in the Kingdom of Saudi Arabia reported that 1743 Saudis and 6064 non-Saudi nationals were living with HIV and approximately 67% of the cases were located in Jeddah, Riyadh and Dammam.[3] The GI tract is a common site for clinical expression of HIV.[4] Virtually all opportunistic infections (OIs) happen when the CD4+ T cell count is less than 200/mm^3^ and subsequently responds to immune reconstitution facilitated by highly active antiretroviral therapy (HAART).[4] Before the advent of HAART, most estimates suggested that 50–93% of the patients with HIV disease present with significant GI complaints during the course of illness.[5]

## PATHOPHYSIOLOGICAL ASPECTS OF HIV INFECTION

HIV has diverse ways of transmission. However, the most common mode is sexual transmission across the genital mucosa,[6] the main determinant of transmission being the viral load.[7] Initial HIV infection happens when the virion binds to a specific combination of receptors located on the host cell. CD4+ lymphocytes and macrophages provide as primary cellular targets of HIV.[8] Subsequent investigations have shown that a secondary receptor or coreceptor, especially the CCR5 and CXCR4 receptors, is also required to facilitate the process of viral entry.[9,10] Pathogenesis of AIDS is mainly due to the reduction of T-lymphocytes, which possess the CD4 receptor.[11] The end point therefore is the subsequent depletion of CD4+ T lymphocytes leading to a failure of both the cellular and the humoral aspects of the body's immunity leading to significant morbidity and mortality, as seen through the natural course of the disease.[12] Consequently, meticulous determination of the CD4+ T lymphocytes in clinical practice is important in the evaluation of the immune system of HIV-infected patients.[13] Progressive immunosuppression is linked to an increase in the prevalence of GI features.[14] The spectrum of GI OIs ranges from oral lesions of Candidiasis, various lesions of viral infections, hepatobiliary lesions and pancreatitis to anorectal lesions.

## ORAL LESIONS

Oral Candidiasis (thrush) manifests in a majority of patients with advanced HIV. Lesions usually present when the CD4 count is less than 200/mm^3^. The lesion presents with oral pain, ageusia and dysphagia. The creamy white lesions are located on the tongue surface and on the buccal mucosa. Occasionally, it may be asymptomatic. Diagnosis is reached by clinical examination and demonstration of yeast forms and pseudohyphae on potassium hydroxide (KOH) preparation. Culture of the scrapping will be useful in identifying the species of the yeast responsible for the infection. Painful oral lesions are caused by Herpes simplex virus (HSV). They show common vesicles that breakdown to form ulcers and have a tendency to be severe and remain for a longer duration in patients with advanced HIV as compared with the normal population.[4] Currently, viral cultures, HSV DNA polymerase chain reactions and HSV serological antigen studies are strongly recommended to ascertain the diagnosis in HIV-positive patients.[15] Lesions usually respond to oral acyclovir, famciclovir or valacyclovir prescribed over a period of 5–10 days. Intravenous acyclovir is however recommended for severe mucocutaneous herpes infection. Acyclovir-resistant strains, as evident by failure of response within 7–10 days, should be managed with foscarnet, which is the drug of choice.[16]

## ESOPHAGEAL LESIONS

Patients with these lesions present with dysphagia, odynophagia or both. Esophagitis is common in advanced disease; at least one-third of the patients will suffer from eosophageal disease. Patients with eosophagitis are usually symptomatic.[17] Candidal eosophagitis is the most frequent,[18,19] occurring either alone or in association with other opportunistic pathogens,[18,20,21] namely Cytomegalovirus (CMV), HSV and ***Mycobacterium avium intercellulare*** (MAI). CMV and Candida coexist in up to 20% of the patients with eosophagitis.[22] The most common viral pathogen causing eosophageal disease is CMV, identified in 10–40% of endoscopic biopsies of eosophageal lesions.[23] Others occurring less commonly are Epstein-Barr virus, HSV and Papovavirus[22,24] and human Herpes virus 6 (HHV-6).[25] MAI causes direct eosophageal infection. Rarely, protozoal causes of eosophagitis include ***Cryptosporidium parvum***, Leishmania spp and ***Pneumocystis jirovecii/carinii***, a fungal species, formerly classified among protozoa.[26–28] Candidal and HSV eosophagitis are predominantly documented in patients with CD4 counts less than 200/mm^3^ whereas CMV is almost exclusively seen with CD4 counts below 100/mm^3^.[11] In the assessment of the condition, double-contrast barium swallow may show multiple filling defects but endoscopy with biopsy is the best method of diagnosis. Given the preponderance of Candidal infection and the classic examination findings, empirical treatment with either oral fluconazole or itraconazole is indicated. Both have a superior efficacy over ketoconazole. A randomized trial demonstrated endoscopic cure rates of 91% vs 52% for fluconazole and ketonazole, respectively.[29] In addition, increasing the doses of micafungin are as effective as fluconazole.[30] Refractory infection will require amphotericin B. In addition, it is advisable to avoid ketoconazole in view of drug interactions with other medications employed in the management of AIDS. Herpes eosophagitis responds to acyclovir. Foscarnet is active against acyclovir-resistant herpes simplex.[16] Established infections with CMV usually respond to a course of ganciclovir or forscarnet followed by oral valganciclovir for 21–28 days or until resolution.[31]

## HEPATOBILIARY DISORDERS

Most patients will experience hepatobiliary symptoms along the course of their illness. MAI is the most common opportunistic pathogen demonstrated in tissues obtained from liver biopsy, accounting for 38% of the diagnosis in a series.[32] Infection usually occurs late when the CD4 counts are less than 50 cells/mm^3^. Hence, the symptomatology is mainly of the systemized illness of disseminated disease[33] although elevation of alkaline phosphatase is typical. The long-term outcome is poor in view of the advanced disease although antimicrobials may produce an initial response. MAI involvement should be distinguished from infections with ***M. tuberculosis***. Extrapulmonary tuberculosis occurs in over 50% of the patients with AIDS.[33] ***M. tuberculosis*** occurs at an early stage of the illness and histology from liver tissue may reveal well-formed caseous granulomatous lesions. Treatment for both typical and atypical tuberculosis should be guided by antimicrobial sensitivities from tissue cultures. Fungi may involve the liver with disseminated disease. Features are mainly with unexplained fever, hepatomegaly and elevated alkaline phosphatase. Fungal abscesses are rarely documented with imaging studies. Hepatic involvement occurs commonly with hematogenous dissemination, as seen with Cryptococcaemia. Less commonly, infections are due to Candida, Coccidioides and Histoplasma. Outcomes of the infection are poor due to disseminated illness and response to extended antifungal therapy is unpredictable. ***Pneumocystis jirovecii (carinii)*** is a fairly common hepatic pathogen in AIDS patients. Involvement occurs in about 38% of the patients.[34] The organism is demonstrable on silver stain, with a characteristic foamy eosinophilic exudate on liver biopsy.[35] Response to parenteral high doses of cotrimoxazole and pentamidine were reported. CMV and Cryptosporidium are associated with acalculous cholecystitis in patients with advanced AIDS.[36] These patients are usually young and present with right upper quadrant pain and abnormal liver profile. Biliary cryptosporidiosis is the most common extraintestinal manifestation of the infection.[37] Clinical features are right upper quadrant pain, nausea, vomiting and fever accompanied by elevated alkaline phosphatase levels. These patients have lower CD4 counts.[38]

## PANCREATITIS

The incidence of pancreatitis in patients with AIDS is estimated to be between 4% and 22%.[39] It presents with abdominal pain in patients with advanced HIV disease, occurring because of diverse reasons. Apart from infections, drugs and neoplasia are also responsible for acute pancreatitis in AIDS patients. Possible infectious causes are numerous and include, in decreasing order of occurrence, CMV,[40] ***M. tuberculosis***,[41] ***M. avium***,[39] Cryptococcus,[42] and HSV.[43] Other infectious causes of pancreatitis in AIDS patients are Candida, ***P. carinii, Toxoplama gondii*** and ***Leishmania donovani***.[44] In assessing the patients, there should be a high index of suspicion when given a setting of HIV disease plus abdominal pain and elevated amylase. Fine needle aspiration should be employed for appropriate diagnosis and treatment.

## PERITONITIS

Infectious peritonitis in the absence of bowel perforation is being recognized in patients with HIV infection. The clinicians should have a low threshold for consideration of peritonitis even in the absence of liver cirrhosis or peritoneal dialysis, as was found in infection with the ***M. avium*** complex[45] in an AIDS patient. It has also been shown that HIV-infected cirrhotic patients tend to have a higher rate of mortality and higher bacteriological isolates, mainly ***Streptococcus pneumoniae*** infection, than non-HIV cirrhotic patients.[46] Other documented infectious causes are Histoplasma,[47] tuberculosis[48] and Cryptococcus.[49]

## APPENDICITIS

The condition in AIDS may occur from faecolith, lymphoid hyperplasia or from infections such as acute CMV infection[50] and mycobacterial infection.[51] Clinical presentation is characterized by right lower quadrant pain, which is associated with low to normal white blood cells.[52] However, before surgical treatment a computed tomography scan or laparoscopy should be arranged for[53] because OIs such as typhlitis may mimic appendicitis.[54]

## SMALL AND LARGE BOWEL LESIONS

Abdominal pain and diarrhea are common in HIV disease. Diarrhea is, however, the most common GI symptom in HIV/AIDS. Prevalence ranges from 0.9% to 14%.[55] Significantly, prevalence is highest in homosexual men and individuals with lower CD4 counts.[56] Generally, CMV infection is the most common OI of the bowel. A wide variety of protozoal, viral and bacterial pathogens are responsible for diarrhea in AIDS patients. MAI is a unique agent of diarrhea in AIDS patients whereas Cryptosporidium causes self-limiting diarrheal illness in healthy hosts.[36,37] It is accompanied by chronic diarrhea in immunosuppressed patients.[57] The manifestations of symptoms by enteric pathogen depend on the degree of immunodeficiency judging by the CD4 cell counts. MAI and CMV are seen in the setting of low CD4 counts of < 100/mm^3^.[58,59] However, sequel to the availability of HAART and antimicrobial prophylaxis, diarrheal illnesses now also results from both ***Clostridium difficile*** colitis and other side effects of drugs.[60] Sexually transmitted diarrheal pathogens are encountered in patients with multiple sexual partners or receptive anal sex.[61] Initial evaluation should include stool smears and cultures for enteric bacteria. The sample is also sent for ***C. difficile*** toxin in the setting of antibiotic usage. It is encouraged to send at least three stool specimens for ova and parasites and acid fast bacilli smear and tuberculosis cultures. However, it has been shown from previous studies than isolation of pathogens is more likely from watery than from formed stools.[62,63] Further assessment requires the utilization of sigmoidoscopy to identify, for instance, CMV infection. Colonoscopy is essential for isolated right colonic CMV.[64] Specific therapy is then directed to the enteric pathogen detected. Recurrent infections with Salmonella, Shigella, Campylobacter and Isospora will require administration of alternating antibiotics.

## ANORECTAL LESIONS

The immunosuppressed patient with AIDS is at an increased risk of abscesses, fistulae, fissure and human Papillomavirus infection.[65,66] The prevalence of anorectal disease among homosexual male patients is high. Fifty-five percent of the 180 consecutive HIV-seropositive patients with anorectal complaints in Chicago were found to be homosexual and bisexual males.[67] Management requires the combination of medical and surgical interventions.

## CONCLUSION

The advent of HAART has significantly changed the spectrum of HIV disease, including the pattern of GI OIs. HAART has led to less immunocompromised settings, effective prophylaxis and the effects of immune reconstitution. The physician should be aware of obvious and subtle presentations of GI manifestations as this will remain a challenge in view of the effect of HAART on patient survival.
